# Preparation and Characterization of Two Immunogens and Production of Polyclonal Antibody with High Affinity and Specificity for Darunavir

**DOI:** 10.3390/molecules25184075

**Published:** 2020-09-07

**Authors:** Ibrahim A. Darwish, Abdulrahman A. Almehizia, Awwad A. Radwan, Rashed N. Herqash

**Affiliations:** 1 Department of Pharmaceutical Chemistry, College of Pharmacy, King Saud University, P.O. Box 2457, Riyadh 11451, Saudi Arabia; mehizia@ksu.edu.sa; 2 Kayyali Chair for Pharmaceutical Industries, Department of Pharmaceutics, College of Pharmacy, King Saud University, P.O. Box 2460, Riyadh 11451, Saudi Arabia; dhna_2001@hotmail.com; 3 Medicinal Aromatic and Poisonous Plant Research Centre, College of Pharmacy, King Saud University, P.O. Box 2457, Riyadh 11451, Saudi Arabia; rherqash@ksu.edu.sa

**Keywords:** darunavir, human immunodeficiency virus, polyclonal antibody, immunoassay, therapeutic drug monitoring

## Abstract

Darunavir (DRV) is a potent antiviral drug used for treatment of infections with human immunodeficiency virus (HIV). Effective and safe treatment with DRV requires its therapeutic drug monitoring (TDM) in patient’s plasma during therapy. To support TDM of DRV, a specific antibody with high affinity is required in order to develop a sensitive immunoassay for the accurate determination of DRV in plasma. In this study, two new and different immunogens were prepared and characterized. These immunogens were the DRV conjugates with keyhole limpet hemocyanin (KLH) protein. The first immunogen (DRV-KLH) was prepared by zero-length direct linking of DRV via its aromatic amino group with the tyrosine amino acid residues of KLH by diazotization/coupling reaction. The second immunogen (G-DRV-KLH) was prepared by conjugation of the N-glutaryl derivative of DRV (G-DRV) with KLH. The 5-carbon atoms-spacing G-DRV hapten was synthesized by reaction of DRV via its aromatic amino group with glutaric anhydride. The reaction was monitored by HPLC and the chemical structure of G-DRV was confirmed by mass, ^1^H-NMR, and ^13^C-NMR spectroscopic techniques. The hapten (G-DRV) was linked to the KLH protein by water-soluble 1-ethyl-3-(3-dimethylaminopropyl) carbodiimide (EDC) coupling procedure. The pertinence of the coupling reactions of haptens to protein was confirmed, and the immunogens were characterized by ultraviolet (UV) spectrophotometry. Both DRV-KLH and G-DRV-KLH were used for the immunization of animals and the animal’s antiserum that showed the highest affinity was selected. The collected antiserum (polyclonal antibody) had very high affinity to DRV (IC_50_ value = 0.2 ng mL^−1^; defining IC_50_ as the DRV concentration that can inhibit antibody binding by 50% of its maximum binding) and high specificity to DRV among other drugs used in the combination therapy with DRV. Cumulative results from direct and competitive enzyme-linked immunosorbent assay (ELISA) using this polyclonal antibody proved that the immunogens were highly antigenic and elicited a specific polyclonal antibody. The produced polyclonal antibody is valuable for the development of highly sensitive and selective immunoassays for TDM of DRV.

## 1. Introduction

The acquired immunodeficiency syndrome (AIDS) refers to a disease of the immune system caused by infection with human immunodeficiency virus (HIV). AIDS is the most advanced stage of HIV infection and it was discovered in the early 1980s [1]. HIV attacks the T lymphocyte cells (CD4 cells) of the immune system, making the body sensitive to life-threatening infections. Two related types of HIV have been identified: HIV-1 and HIV-2 [2]. The United States Food and Drug Administration (US-FDA) approved zidovudine as the first antiretroviral drug for treatment of AIDS [3,4]; however, its therapeutic impacts were disappointing because the virus developed resistance to the drug. Subsequently, a more effective class of drugs were introduced for treatment of HIV infections; this class was the protease inhibitor drugs [5,6]. The therapeutic potencies and safeness of protease inhibitor drugs led to a dramatic decline in the morbidity and mortality among HIV-infected patients [7].

**Figure 1:**
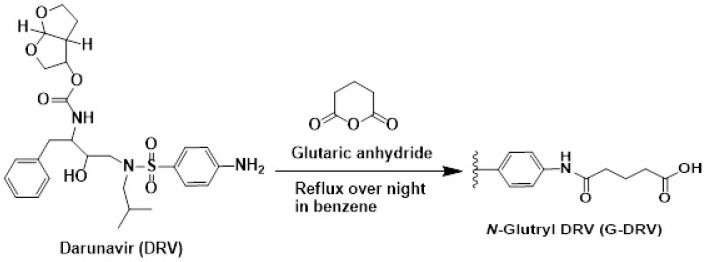
Reaction mechanism for the synthesis of *N*-glutaryl darunavir (DRV).

Darunavir (DRV) [(3aS,4R,6aR)-2,3,3a,4,5,6a-hexahydrofuro [2–3-b]furan-4-yl] *N*-[(2S,3R)-4-[(4-aminophenyl)sulfonyl-(2-methylpropyl)amino]-3-hydroxy-1-phenylbutan-2-yl]carbamate (Figure 1) is a synthetic non-peptide protease inhibitor developed in 1998 [8]. In June 2006, DRV was first approved by the FDA for treatment of resistant type-1 of HIV [9]. In July 2016, the FDA expanded the approval for its use in pregnant women with HIV infection [10]. DRV prevents the replication of the HIV virus by inhibiting the catalytic activity of the HIV-1 protease enzyme. It is unique among currently available protease inhibitors because it maintains antiretroviral activity against a variety of multi-drug-resistant HIV strains [11].

Although the successful therapeutic results of DRV, its pharmacokinetics showed inter- and intra-individual variability, with both consistent concentration–efficacy and concentration–toxicity relationships [12]. In spite of these pharmacokinetic variability, the dose should be adjusted as per each patient’s status and therapeutic drug monitoring (TDM) for DRV should be conducted. The TDM is helpful in achieving the highest therapeutic benefits and preventing any potentially fatal complications. The existing methods for determination of DRV in patient samples are mostly liquid chromatography-coupled with tandem mass spectrometric detector (LC-MS/MS) [13,14]. These methods are time-consuming, have a limited throughput and require tedious extraction procedures that negatively affect the accuracy of the results [13,14,15]. Moreover, these instruments are very expensive to be available in most laboratories in third-world countries. Therefore, alternative techniques with adequate selectivity and sensitivity with more simplicity, lower cost, and higher throughput are essential. In previous studies [16,17,18,19,20], different immunoassays have been successfully developed for TDM of various drugs. These methods offered great selectivity, high sensitivity, high throughput, and low cost. In literature, no immunoassay exists for the determination of DRV in plasma samples. The present study describes, for the first time, the preparation of two different immunogens for DRV and production of an antibody with high affinity and specificity for DRV. This antibody is useful for the development of immunoassay systems for the determination of DRV in plasma samples.

## 2. Results

### 2.1. Synthesis and Structure Confirmation of DRV Hapten (N-Glutaryl-DRV)

**Figure 2:**
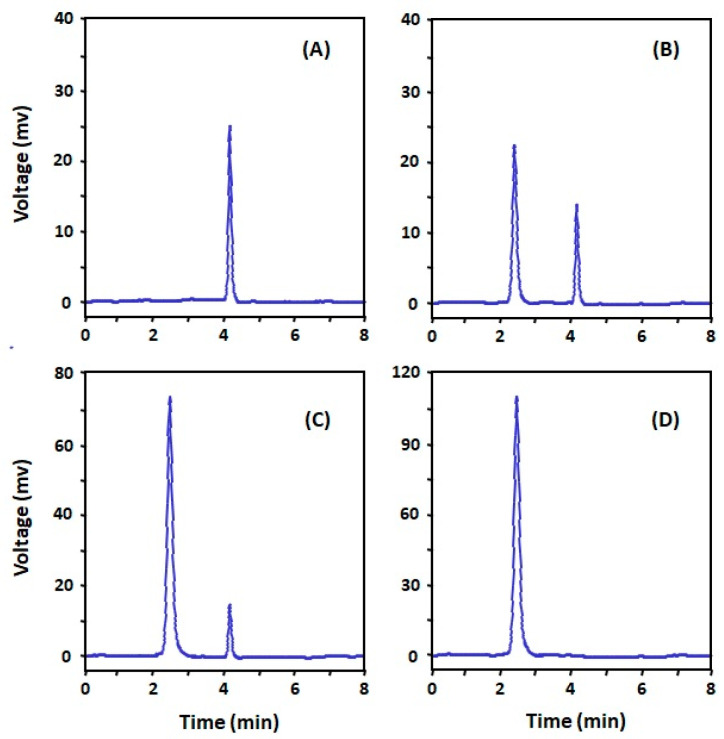
Chromatogram of standard DRV (**A**), reaction mixture of DRV with glutaric anhydride after 4 h (**B**), the same reaction mixture after 20 h (**C**), and the final product of the reaction, *N*-glutaryl-DRV (**D**). Chromatographic conditions were: reversed phase column (Nucleosil C8, 150 × 4.6 mm, 5 µm), isocratic elution by a mobile phase which consists of acetonitrile:water (50:50, *v*/*v*), the flow rate was set at 1 mL min^−1^, and the detection was UV at 266 nm.

**Figure 3:**
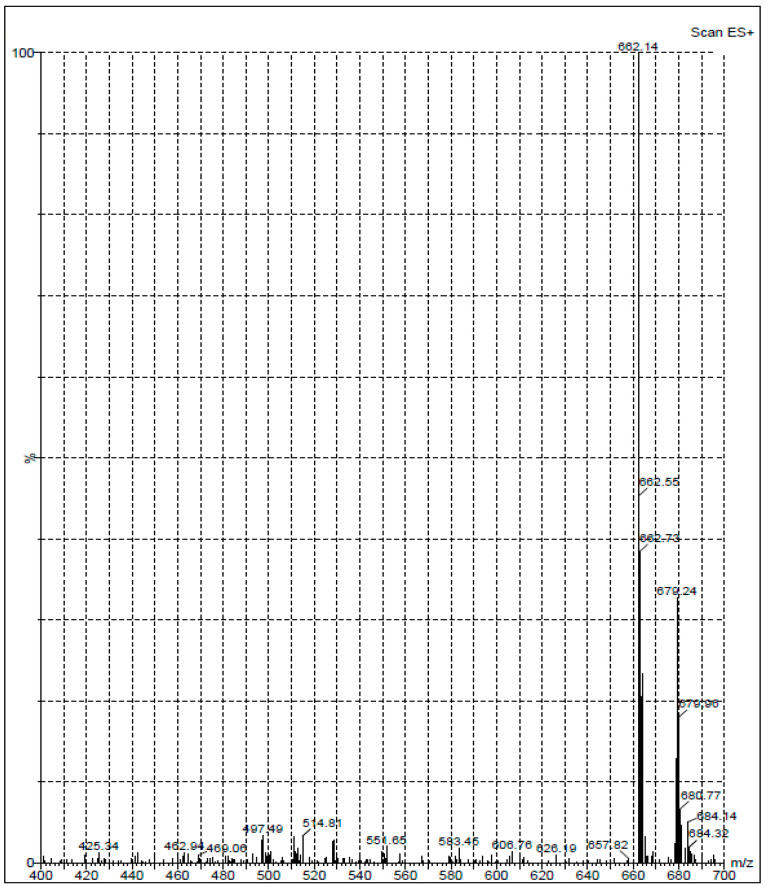
Total ion scan of the synthesized *N*-glutaryl DRV.

**Figure 4:**
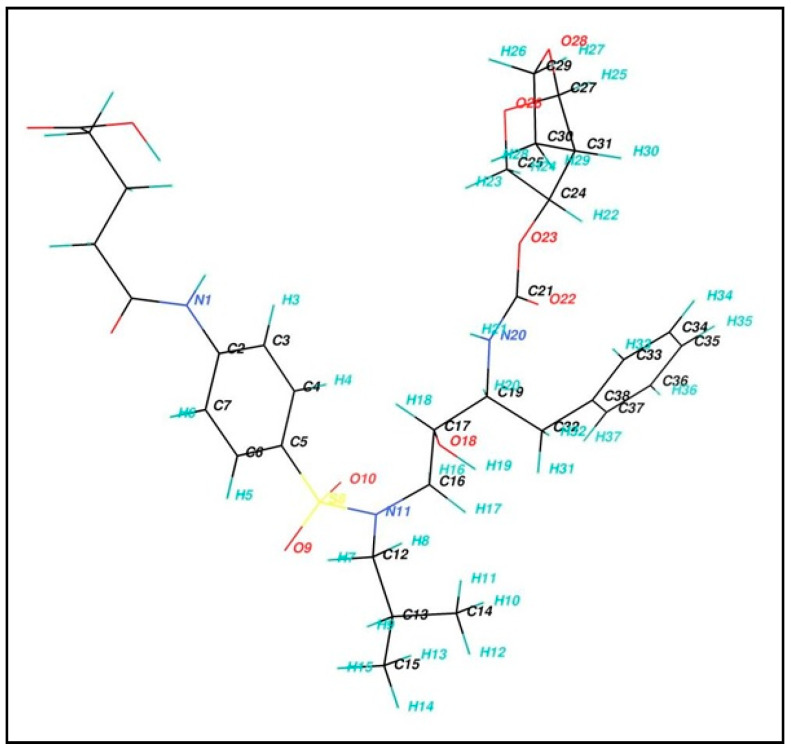
Chemical structure of the synthesized *N*-glutaryl DRV with assigned carbon and hydrogen atoms.

Generation of antibodies against small molecules, such as DRV, is difficult because of their inability to elicit immune response and produce antibodies. Therefore, it was necessary to modify the DRV molecule by its coupling with some macromolecules (e.g., proteins) in order to produce a stable DRV-protein conjugate. It was possible to directly couple DRV with a protein via the aromatic amino group of DRV by diazotization/coupling procedure. The procedure involved diazotization of the DRV aromatic amino group then linking to the tyrosine amino acid residues of the proteins [21]. The insertion of a “spacer” between the hapten molecule and the proteins has been proved to increase the specificity of the produced antibody [22]. Therefore, we aimed to insert a glutaryl moiety as a “spacer” between DRV and the protein. Glutaryl moiety was inserted into the DRV structure through its primary aromatic amino group. The *N*-glutaryl derivative of DRV (G-DRV) was synthesized by the reaction of DRV with glutaric anhydride (Figure 1), and the reaction was monitored by HPLC. The chromatogram of the reaction mixture (Figure 2) showed two peaks. The firstly eluted peak (at a retention time of 2.55 ± 0.27 min) was thought to be the glutaryl-DRV derivative. The other peak eluted at a retention time similar to that of DRV (at 4.1 ± 0.20 min) indicating that this peak corresponds to the unreacted DRV. LC/MS spectrometry for the former peak gave M + 1 peak at 662.14 indicating that it was the G-DRV (Figure 3). At the end of the reaction, the chemical structure of the product (G-DRV) was confirmed by ^1^H-NMR and ^13^C spectrometry (Figure 3 and Figure 4).

**Figure 5:**
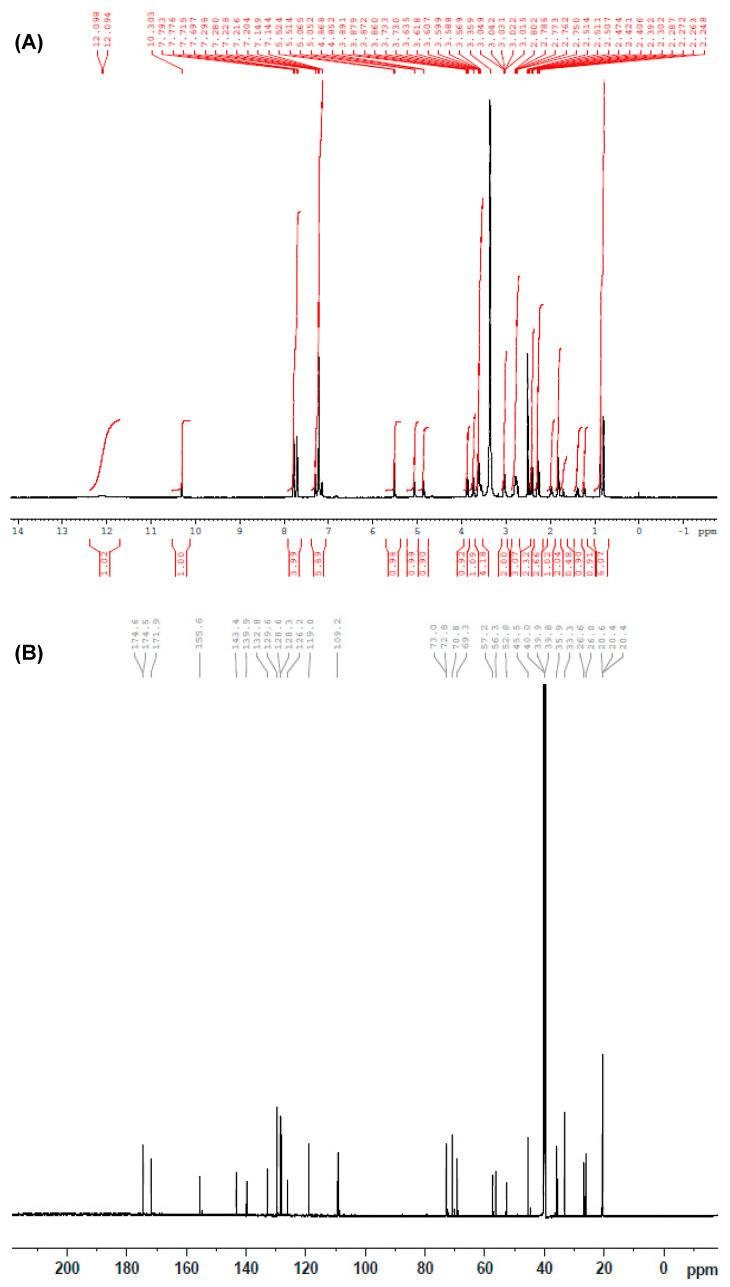
^1^H-NMR (**A**) and ^13^C (**B**) spectra of glutaryl-DRV derivative. NMR spectra were scanned in DMSO-d6 on a Bruker NMR spectrometer operating at 500 MHz for ^1^H and 176 MHz for ^13^C. Chemical shifts are expressed in δ-values (ppm) relative to tetramethyl silane (TMS) as an internal standard. D_2_O was added to confirm the exchangeable protons.

The ^1^H-NMR spectrum (DMSO-*d*_6_) of the product is given in Figure 5A. δ 2.39–2.47 (m, 4H, COCH_2_CH_2_CH_2_COOH) and δ 1.95 (m, 2H, COCH_2_CH_2_CH_2_COOH) of glutaryl moiety confirmed the introduction of the glutaryl moiety into the DRV structure (Table 1). The ^13^C-NMR spectrum of glutaryl derivative of DRV is given in Figure 5B. Appearance of two signals at downfield; δ 171.96 (N-COCH_2_CH_2_CH_2_COOH) and δ 174.62 (N-COCH_2_CH_2_CH_2_COOH) of the glutaryl DRV indicate the presence of C=O carboxylic acid and C=O amide carbons of the glutaryl moiety.

### 2.2. Preparation and Characterization of Protein Conjugates

**Figure 6:**
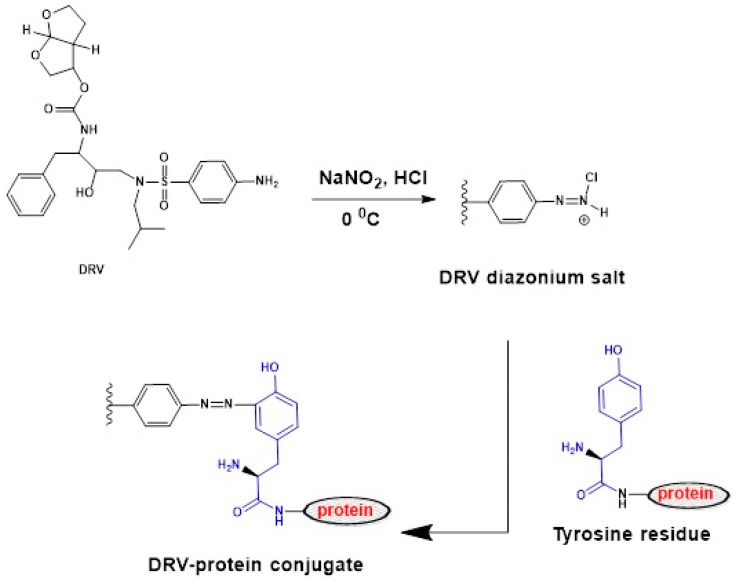
Preparation of DRV-protein conjugates by diazotization/coupling reaction with protein (bovine serum albumin (BSA) and keyhole limpet hemocyanin (KLH)) via their tyrosine amino acid residues.

**Figure 7:**
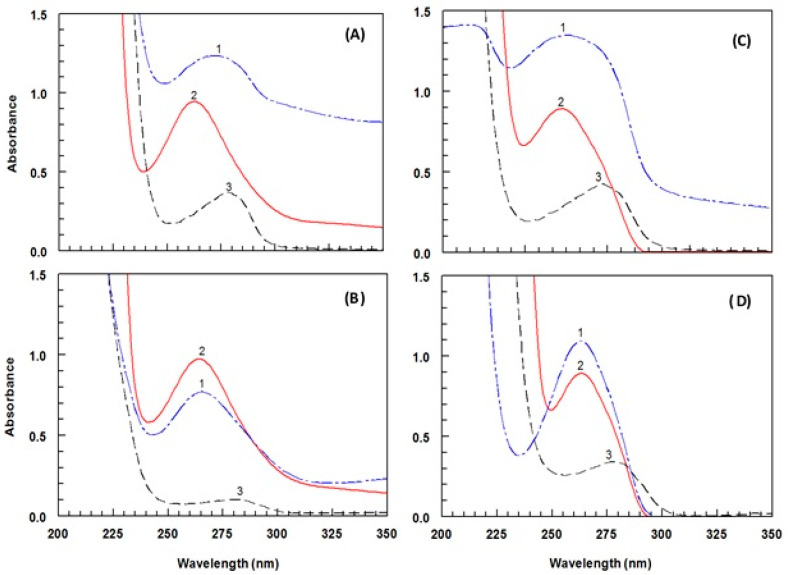
UV absorption spectra for characterization of DRV-BSA (**A**), DRV-KLH (**B**), G-DRV-BSA (**C**), and G-DRV-KLH (**D**) conjugates. Spectra were DRV-protein or G-DRV-protein conjugate (1), DRV (2) and unconjugated protein (3). Concentrations of DRV and G-DRV were 100 µg mL^−1^ and equal concentrations of DRV-protein, G-DRV-protein, BSA, and KLH (500 µg mL^−1^) prepared in 50 mM PBS (pH 7.4).

In this study, two groups of protein conjugates were prepared. The first group was prepared by direct linking of DRV with each of bovine serum albumin (BSA) and keyhole limpet hemocyanin (KLH) proteins by diazotization/coupling reaction as illustrated in Figure 6. The nitrosation of primary aromatic amine with nitrous acid (generated in situ from sodium nitrite and hydrochloric acid) led to the formation of diazonium salt, which could be coupled with the protein via its tyrosine amino acid residues [23]. The second group of conjugates was prepared by linking the G-DRV, via its COOH group, to each of KLH and BSA by carbodiimide method [24]. The desired conjugation reaction proceeded being catalyzed by H^+^; however, the carrier protein is more reactive at higher (less acidic) pH where there is a dissociation of the neutrality to compromise and which provided the most favorable conditions for the reaction [24]. Formation of the DRV-protein and G-DRV-protein conjugates was confirmed by the UV-spectrophotometric analysis for the protein conjugates, haptens, and the unconjugated proteins under the same conditions. The spectra obtained in the case of the DRV-protein and G-DRV-protein conjugates are illustrated in Figure 7. It is obvious that the conjugates showed higher absorbances than those of unconjugated proteins at their maximum absorption peaks. These hyperchromic effects with blue shifts in the absorption spectra of the conjugates compared with the unconjugated proteins were evident for the successful conjugation of DRV and G-DRV with the proteins and formation of the conjugates.

### 2.3. Production of Anti-DRV Polyclonal Antibody

**Figure 8:**
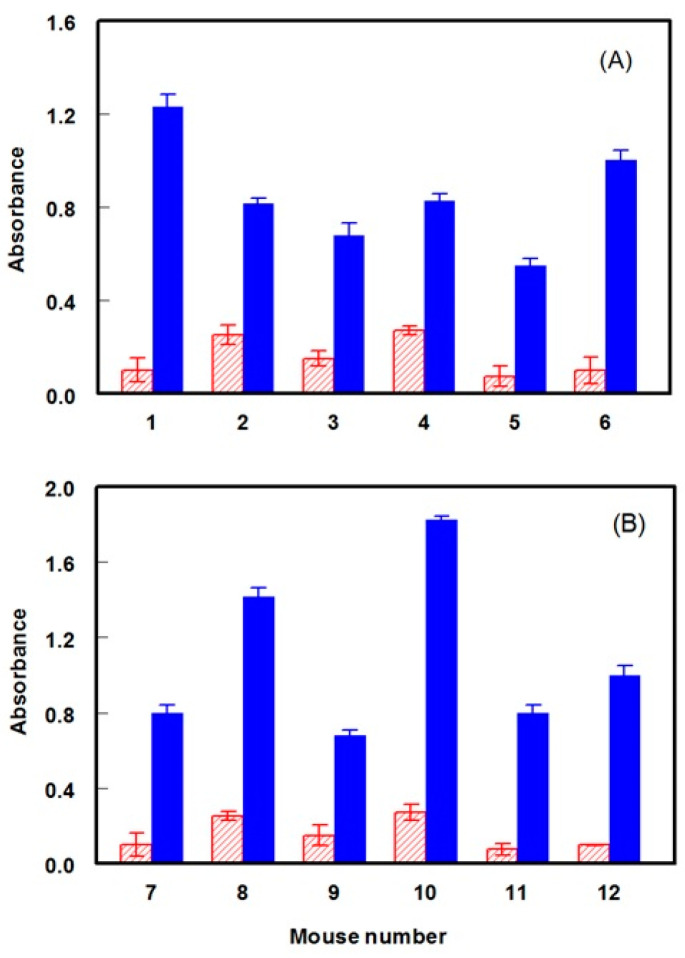
Immune response of mice immunized with DRV-KLH (**A**) and with G-DRV-KLH (**B**). In panel A, microwell plates were coated with BSA (red striped bars) and with DRV-BSA conjugate (blue solid bars). In panel B, microwell plates were coated with BSA (striped bars) and with G-DRV-BSA conjugate (solid bars).

Due to the high immunogenicity of KLH [24], DRV-KLH and G-DRV-KLH conjugates were used as immunogens for production of anti-DRV polyclonal antibody and the DRV-BSA and G-DRV-BSA conjugates for immobilization onto the microwell plates as solid-phases in the ELISA used for screening of antisera of immunized animals. Two groups of mice (BALB/c white female mice of 8 weeks old) were subjected to immunization (six mice were used for each group). Mice of the first group were given numbers from 1 to 6 and those of the second group were given numbers from 7 to 12. The first group was immunized with DRV-KLH conjugate and the second group was immunized with G-DRV-KLH conjugate. In order to ensure the immune response of the mice to the immunization with DRV-KLH and G-DRV-KLH conjugates and to confirm that the animals were sufficiently immunized, serum samples were collected from each animal after 7 days from the fifth booster injection (the sixth immunization injection). The collected serum samples were analyzed by direct ELISA [23]. As shown in Figure 8, the absorbances for all antisera obtained when DRV-BSA and G-DRV-BSA conjugates were used as immobilized antigens were higher than those obtained when unconjugated BSA protein was used as immobilized antigen. To assess and compare if the antibody responses obtained in both groups (Figure 8) are statistically different, statistical analysis for the results was conducted taking the mean ± standard deviation of the six mice per group. The average absorbance values were found to be 0.81 ± 0.20 and 1.12 ± 0.17 for the groups immunized with DRV-KLH and G-DRV-KLH, respectively. This result indicated that the DRV modification with the spacer (G-DRV-KLH) was required for higher (statistically significant) immune responses compared to group immunized with directly linked DRV-KLH. It is wise to mention that assay plates were coated with BSA conjugates of DRV to exclude the fraction of polyclonal antibodies recognizing the KLH part of the immunogen.

These data indicate the immune response of all mice to the immunization and the specific recognition of the evoked antibodies for DRV residues in the immobilized DRV-BSA and G-DRV-BSA conjugates, but not for recognition of the BSA molecule. These cumulative results indicate that successful immunizations of the mice have been attained. The observed small absorbance values when BSA protein was used as an immobilized antigen were attributed to the nonspecific binding of the mouse immunoglobulins to the plate wells. This nonspecific binding can be overcome when a proper optimization of the assay conditions (e.g., concentrations of reagents, etc.) is carried out.

### 2.4. Selection of the Antibody with the Highest Affinity to DRV

**Figure 9:**
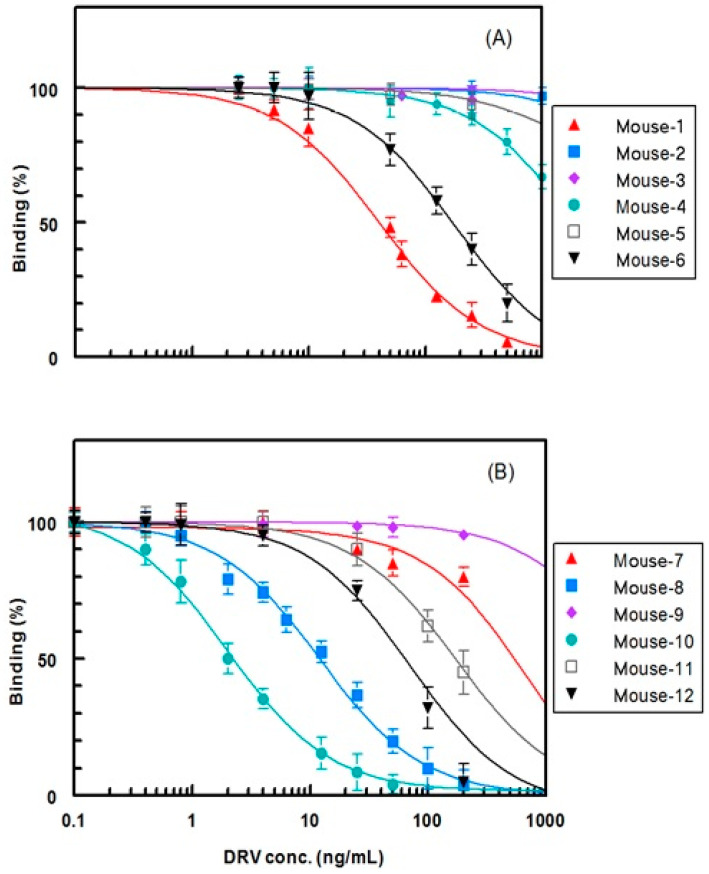
Competitive ELISA for determination of the affinity of antisera collected from mice immunized with DRV-KLH (**A**) and with G-DRV-KLH (**B**) to DRV. Microwell plates were coated with DRV-BSA (**A**) and with G-DRV-BSA (**B**).

In order to select the most convenient antiserum, the affinity of each antiserum to DRV was measured by the competitive ELISA [18]. IC_50_ values were used as measures for the affinity; IC_50_ was defined as the concentration of DRV that causes 50% inhibition from the maximum binding of the antiserum to the coated conjugate (DRV-BSA or G-DRV-BSA). The binding percentage (B) was calculated using the formula: B = (Ac/A0) × 100; where Ac is the absorbance obtained at a definite concentration of DRV, and A0 is the absorbance of blank wells (maximum absorbance at zero concentration of DRV). As shown in Figure 9 and Table 2, the antisera collected from the mice of the second group gave higher affinities (lower IC_50_ values) than those collected from the mice of the first group. The antiserum of mouse 10 gave the highest affinity (lowest IC_50_; 2 ng mL^−1^) to DRV. Therefore, this antiserum of mouse 10 was selected for further investigations and was called anti-DRV antibody thereafter.

It is worth mentioning that the selection of a spacing linker between the hapten molecule and protein during the design of the immunogen is an important issue. Some studies [25,26] suggested that the linker should be long and flexible enough to ensure that the hapten molecule connected to the protein could have sufficient spatial freedom to be exposed to the lymphocyte cells and ultimately produce hapten-recognizing antibodies. The suggested optimum length range of linkers should be 3-8 carbon atoms. A too short linker may cause spatial steric hindrance within the protein molecule, affecting its folding and may hide the hapten residues inside. A too long linker may make the hapten molecule far away from the immunogenic protein and thus the induced antibody molecules will be mostly specific to the protein rather than the desired hapten molecules. Therefore, the glutaryl moiety with five carbon atoms was considered in our present study. The higher affinity of the antisera induced using G-DRV-KLH compared with those induced by DRV-KLH confirm the reliability of our design for the immunogens.

### 2.5. Effect of Type of Plate-Coating Conjugate on the Affinity and Specificity of Anti-DRV Antibody

**Figure 10:**
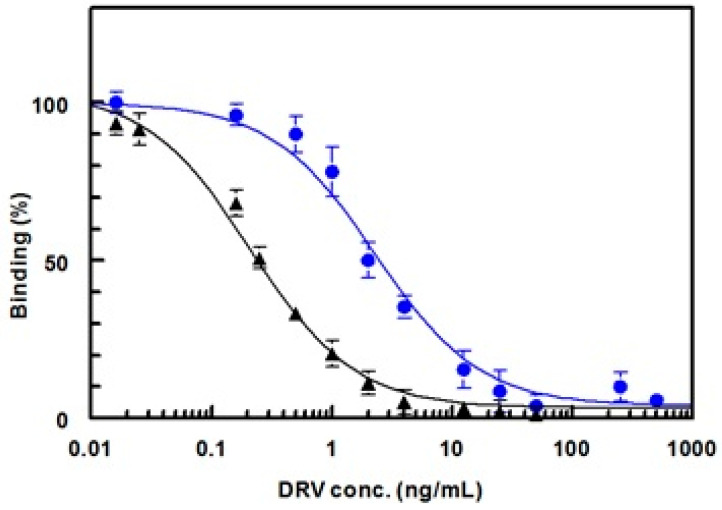
Affinity of anti-DRV antibody to DRV when microwell plates were coated with DRV-BSA (▲) and with G-DRV-BSA (●) conjugates.

In the competitive ELISA used in this study, DRV was quantified by its ability to inhibit the binding of its antibody to the coated conjugate (DRV-BSA or G-DRV-BSA) in a competitive fashion. In this approach, the affinity of the antibody to its analyte is the most important factor that determines the sensitivity of the ELISA [22]. To achieve maximum sensitivity for the competitive ELISA, the affinity of the anti-DRV antibody must be preferentially higher to the free DRV (in sample solution) than its affinity to DRV residues in the coated conjugate. In order to select the better conjugate (DRV-BSA or G-DRV-BSA) that provides a maximum sensitivity for the assay, competitive ELISA was carried out for the anti-DRV antibody using both conjugates (DRV-BSA and G-DRV-BSA) for coating onto the microwells, and IC_50_ values were determined in each case. It was found that the affinity of anti-DRV antibody to DRV was significantly increased (i.e., IC_50_ decreased) when DRV-BSA was used as coating antigen, rather than G-DRV-BSA (Figure 10). The IC_50_ values were 0.2 and 2.0 ng mL^−1^ when the DRV-BSA and G-DRV-BSA were used as coating antigens, respectively. This was explained by the fact that the *N*-glutaryl moiety of the G-DRV-BSA conjugate was one of the recognition epitopes for the anti-DRV antibody. Therefore, the affinity of the antibody was significantly directed to the free DRV when DRV-BSA conjugate, that did not contain *N*-glutaryl moiety, was used as coating antigen. Accordingly, DRV-BSA was used for coating onto the microwells of the assay plate in the subsequent experiments that involved the development of an immunoassay for DRV. This high sensitivity (IC_50_ = 0.2 ng mL^−1^) enables the determination of therapeutic concentrations of DRV in plasma; the reported therapeutic concentrations were 1255–7368 ng mL^−1^ after 600 mg twice-daily dosing [27].

**Figure 11:**
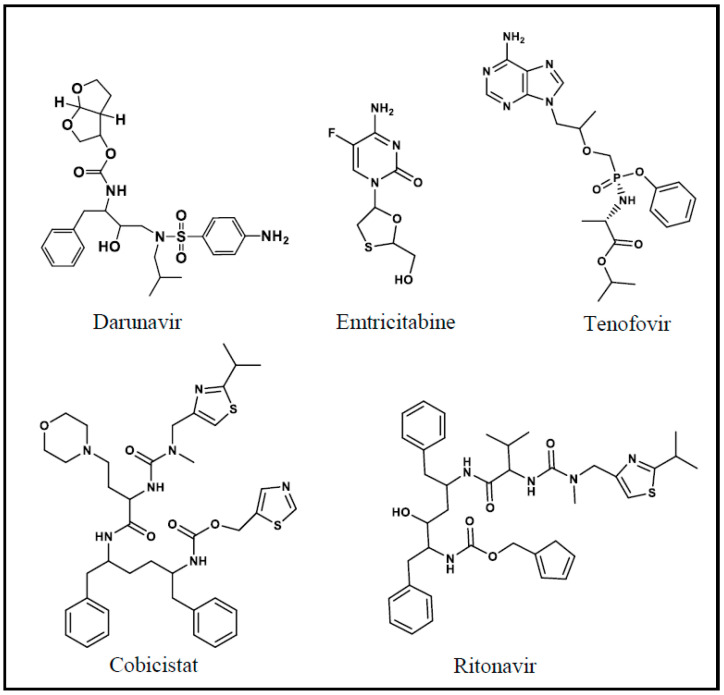
The chemical structures of DRV and other drugs co-administrated with DRV in the combination therapy.

**Figure 12:**
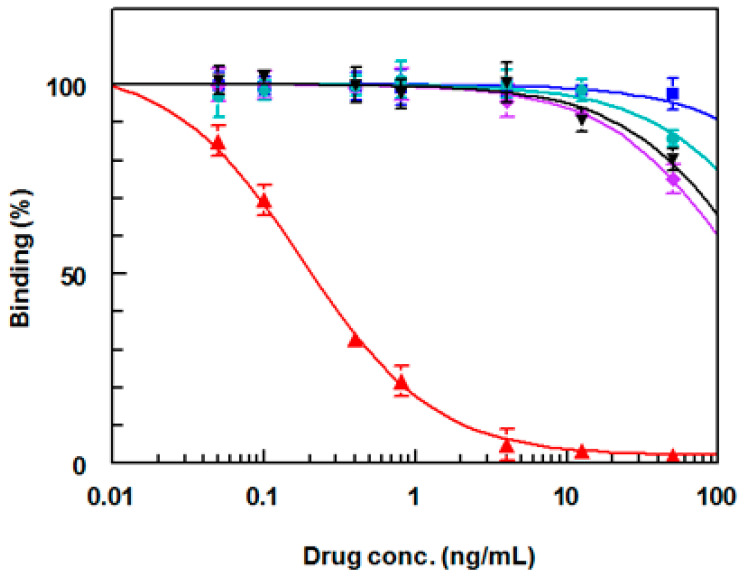
Competitive assay for DRV (▲), ritonavir (♦), cobicistat (▼), tenofovir (●), and emtricitabine (■).

In order to assess the specificity of the anti-DRV antibody, a competitive assay was carried out using various competitors for DRV. These competitors were the drugs that have been reported to be co-administrated with DRV in the combined therapy. These drugs were ritonavir, cobicistat, tenofovir, and emtricitabine [28]. The chemical structures of these drugs are given in Figure 11. It is obvious that the antibody had high affinity as its IC_50_ was 0.2 ng mL^−1^ compared with IC_50_ values of higher than 100 ng mL^−1^ for the other drugs (Figure 12). These data proved that none of these drugs showed any cross reactivity with DRV in the analysis of samples containing DRV and any of these drugs.

### 2.6. Applications of the Anti-DRV Polyclonal Antibody

The aforementioned results, in terms of the affinity and specificity of the anti-DRV antibody, confirmed its valuable use and applications in the development of immunoassay systems for the accurate determination of DRV in the plasma sample. Using this antibody, we optimized and validated the ELISA system [29], and a current work is going to use this antibody in developing two different formats of heterogenous fluoroimmunoassays; microplate-based and KinExA immunosensor-based assays. Optimization and validation of these assays will be published elsewhere.

## 3. Experimental

### 3.1. Instruments

Microplate absorbance reader (ELx808: Bio-Tek Instruments Inc., Winooski, VT, USA). Automatic microplate strip washer (ELx50/8: Bio-Tek Instruments Inc., Winooski, VT, USA). Ultrasonic sonicator cleaning system (X-TRA150H: Elma, England). Refrigerated centrifuge (1-15PK: Sigma Laborzentrifugen GmbH, Osterode, Germany). Incubator (MINI/18: Genlab Ltd. Widnes, United Kingdom). Microprocessor laboratory pH meter (BT-500: Boeco, Hamburg, Germany). Electric digital balance (JB1603-C/FACT: Mettler-Toledo International Inc., Zürich, Switzerland). UV-VIS spectrophotometer (UV-1601 PC: Shimadzu, Kyoto, Japan). Purelab Flex water purification system (ELGA Veolia Ltd., High Wycombe, United Kingdom). HPLC system from Waters controlled by Empower software (Waters Corporation, Milford, Massachusetts, USA) equipped with Waters 1525 binary pump, autosampler Waters 2707, column oven 5CH, UV–VIS detector Waters 2489, and system controller MIL-S800i-V2. Mass spectrometer (Varian TQ 320 GC/MS/MS: Varian, Palo Alto, Santa Clara, CA, USA). Bruker NMR spectrometer (Bruker Corporation, Bruker Daltonik GmbH, Bremen, Germany).

### 3.2. Materials

Darunavir (DRV) was obtained from Parchem Fine & Specialty Chemicals (New York, NY, USA). Glutaric anhydride and 1-ethyl-3-(3-dimethylaminopropyl) carbodiimide hydrochloride (EDC) was purchased from Sigma-Aldrich Co. (St. Louis, MO, USA). Keyhole limpet hemocyanin (KLH) was purchased from Novabiochem Co. (La Jolla, CA, USA). BCA protein assay kit was a product of Pierce Chemical Co., Rockford, IL, USA). Bovine serum albumin (BSA), 3,3′,5,5′-tetramethylbenzidine (TMB) peroxidase substrate, Freund’s adjuvants (complete and incomplete), goat anti-mouse IgG-horseradish peroxidase conjugate, and the dialysis tube were obtained from Sigma-Aldrich Co. (St. Louis, MO, USA). EIA/RIA high-binding microwell plates were purchased from Corning/Costar, Inc. (Cambridge, MA, USA). All other chemicals and solvents used throughout the work were of analytical grade.

### 3.3. Procedures

#### 3.3.1. Synthesis of Hapten (*N*-Glutaryl Darunavir; G-DRV)

Glutaric anhydride solution (57 mg dissolved in 10 mL of benzene was prepared and a quantity of darunavir (274 mg) was added and mixed. The mixture was kept under reflux overnight during which the reaction was monitored by HPLC chromatography for the formation of *N*-glutaryl DRV (G-DRV). The chromatographic conditions were: reversed phase column (Nucleosil C8, 150 × 4.6 mm, 5 μm), a mobile phase that comprised acetonitrile:water (50:50, *v*/*v*) which was pumped isocratically at a flow rate of 1 mL min^−1^. The UV detector was set at 266 nm. After the reaction was completed, the G-DRV was purified by crystallization from ethanol. The purity of the product was confirmed by the HPLC system. Mass, ^1^H-NMR, and ^13^C spectrometric techniques were used to confirm the chemical structure of the purified G-DRV.

#### 3.3.2. Preparation of DRV–Protein Conjugates by Diazotization/Coupling Reaction

Protein (KLH and BSA) solutions (5 mL, 10 mg mL^−1^) were prepared in phosphate buffer (PB: 50 mM, pH 7.2). Solution of DRV (10 mg mL^−1^) was prepared by dissolving 50 mg of DRV in 5 mL of dimethylformamide. To the DRV solution, 0.5 mL 0.5 M HCl was added, followed by drop wise addition of sodium nitrite solution (0.3 mL, 0.1 M in water). The diazotization reaction was performed with stirring at 4 °C for 30 min. Urea (0.9 mg, 15 µmol) was added and the mixture was stirred for 5 min during which the excess nitrous acid produced was decomposed. Then, the reaction mixture was divided into two equal parts. One part was added to KLH solution and the other was added to the BSA solution. The reactions were left to proceed at 4 °C overnight. The residual unreacted DRV molecules were removed from the DRV–protein conjugates by dialysis. The products were collected and the conjugations of DRV to each KLH and BSA were confirmed by protein assay and UV spectral analysis [30].

#### 3.3.3. Preparation of G-DRV–Protein Conjugates by Carbodiimide Method

A quantity (50 mg) of each of the KLH and BSA proteins was dissolved in 5 mL of PB (50 mM, pH 7.2) producing the protein solutions (10 mg/mL). G-DRV (hapten) solution (10 mg mL^−1^) was prepared by dissolving 50 mg of G-DRV in 5 mL of dimethylformamide. To this solution, 100 mg of 1-ethyl-3-(3-dimethylaminopropyl) carbodiimide hydrochloride (EDC) was added, and the pH was rapidly adjusted to 5–5.5 with HCl (0.01 M). After 5 min, the protein (KLH or BSA) solution was added and the pH of the reaction mixture was quickly adjusted to 6.4 and maintained constant for 90 min. The reaction was left overnight in the dark at 4 °C. Dialysis was used to remove residual unreacted hapten (G-DRV) molecules from G-DRV–protein conjugates. The products were collected and the conjugations of G-DRV to each BSA and KLH were confirmed by protein assay and UV spectral analysis [30].

#### 3.3.4. Immunization of Animals and Production of Anti-DRV Polyclonal Antibody

Two groups of mice (white female BALB/c, 8-weeks old) were immunized; six mice for each group. The first group (mouse 1 to mouse 6) was immunized with DRV-KLH conjugate and the second group (mouse 7 to mouse 12) was immunized with G-DRV-KLH conjugate. Mice were kept in polycarbonate plastic cages containing wood shavings as bedding. The food and water were freely available during the experiment. Anesthesia and/or analgesia have not been used in the immunization. The immunization was conducted according to previous procedures [31]. Briefly, the immunogen solutions (1 mg mL^−1^) of each conjugate prepared in phosphate buffer saline (PBS) were mixed with an equal volume of complete Freund’s adjuvant and the mixture was emulsified. Each mouse was subjected to an intraperitoneal injection with 100 μL of the corresponding immunogen solution emulsified in Freund’s complete adjuvant. The mice were subjected to five boosting injections at every 3-week intervals; however, incomplete adjuvant was used instead. After 7 days from the fifth booster injection (6th injection from the first immunization injection), blood samples were collected from the immunized mice in serum-collecting tubes. At the end of the experiments, animals were sacrificed by exposure to CO_2_ gas according to the Guidelines for euthanasia of rodent fetuses and neonates. The blood samples were diluted 10-fold with PBS and centrifuged at 12,000 rpm at 4 °C for 5 min. The sera (supernatants) were collected and the antibody response in each serum was tested by direct ELISA [32]. The affinity of each serum to DRV was determined by a competitive ELISA [18]. The serum that exhibited the highest affinity to DRV was selected to be used as the crude anti-DRV polyclonal antibody.

## 4. Conclusions

In this study, two new immunogens for DRV were synthesized, characterized, and used for the production of polyclonal antibody with high affinity and specificity for DRV. The first immunogen (DRV-KLH) was prepared by zero-length direct linking of DRV to KLH by diazotization/coupling procedures. The second immunogen (G-DRV-KLH) was prepared by linking the *N*-glutaryl derivative of DRV (G-DRV) with KLH by the carbodiimide method. The 5-carbon atoms-spacing G-DRV derivative used for preparation of this immunogen was synthesized by reaction of DRV with glutaric anhydride, and its chemical structure was confirmed by mass, ^1^H- and ^13^C-NMR spectroscopic techniques. Both immunogens were highly antigenic and elicited polyclonal antibodies; however, the antibody produced by the second immunogen had the highest affinity (IC_50_ = 0.2 ng mL^−1^). This antibody was also highly specific to DRV among other drugs used in the combination therapy with DRV, as none of these drugs did not cross react with DRV. Using this polyclonal antibody, a highly sensitive ELISA has been developed [29] and the results proved the potential use of this assay to contribute to the safe and effective treatment with DRV via its TDM in patients. The glutaryl spacer does not have any immunogenicity; however, its introducing between DRV and KLH proteins enhanced the affinity of the evoked polyclonal antibodies to DRV.

## Figures and Tables

**Table 1:** ^1^H-NMR spectral data of *N*-glutaryl derivative of DRV (in DMSO-d6).

Signal	Location (δ)	Shape	Integration	Corresponding Carbon Number
1	0.78	d, *J* = 6.5 Hz	3H	C_14_
2	0.86	d, *J* = 6.5 Hz	3H	C_15_
3	1.42	m	1H	C_13_
4	1.82	m	3H	C_30_,C_31_
5	1.95	m	2H	COCH_2_CH_2_CH_2_COOH
6	2.23–2.30	m	2H	C_32_
7	2.39–2.47	m	4H	COCH_2_CH_2_CH_2_COOH
8	2.75–2.80	m	2H	C_12_
9	3.03	d	2H	C_16_
10	3.56–3.89	m	5H	C_17_, C_19_, C_25_, C_29_
11	4.86	m	1H	C_24_
12	7.05	m	1H	C_27_
13	5.51	s	1H	OCONH-C<
14	7.14–7.29	m	5H	C_6_H_5_-CH_2_-
15	7.74	dd	4H	C_6_H_4_-SO_2_-
16	10.30	s	1H	-NH-CO-glutaryl
17	12.9	s	1H	COOH

**Table 2:** IC_50_ values obtained for the antisera from the mice of the two groups ^a^.

Group 1: Immunized with DRV-KLH		Group 2: Immunized with G-DRV-KLH	
Mouse No.	IC_50_ (ng mL^−1^)	Mouse No.	IC_50_ (ng mL^−1^)
1	40	7	>1000
2	>1000	8	12
3	>1000	9	500
4	>1000	10	2
5	>1000	11	180
6	160	12	60

^a^: IC_50_ is defined as the DRV concentration that cause 50% inhibition of antibody binding to the coated antigen.
